# Body Representations of Internal Pollution: The Risk Perception of the Circulation of Environmental Contaminants in Pregnant and Breastfeeding Women in Spain

**DOI:** 10.3390/ijerph17186544

**Published:** 2020-09-08

**Authors:** Cristina Larrea-Killinger, Araceli Muñoz, Arantza Begueria, Jaume Mascaró-Pons

**Affiliations:** 1 Food Observatory, Department of Social Anthropology, University of Barcelona, 08001 Barcelona, Spain; aracelimunoz67@ub.edu (A.M.); arantzazu.begueria@gmail.com (A.B.); mascaropons@gmail.com (J.M.-P.); 2 CIBER de Epidemiología y Salud Pública (CIBERESP), 28029 Madrid, Spain; 3 School of Social Work, University of Barcelona, 08035 Barcelona, Spain

**Keywords:** pollution, body, pregnancy, breast milk, risk perception, body mapping

## Abstract

In this article, we analyze how pregnant and breastfeeding women perceive the inside of their bodies as well as their thoughts regarding the accumulation and elimination of chemical compounds present in food, and how these are then transmitted to the fetus. We explore different social perceptions of risk regarding the circulation of chemical compounds inside the body using qualitative research based on the technique of body mapping, comprised of women’s figures of their bodies in combination with comments on the figures, food diaries and narratives from in-depth interviews. We examine how these 41 women (21 pregnant and 20 breastfeeding) perceive the body’s internal mechanisms during the stages of pregnancy and breastfeeding, as well as the circulation of chemical contaminants within it. The body mapping technique allowed us to analyze participants’ knowledge of internal pollution, a little-understood process in society. Thanks to these pregnant and breastfeeding women, who made an effort to represent and reflect on these new risks, this study shows that scientists and obstetricians need to collaborate with women in order to better understand and publicize the risks of internal pollution.

## 1. Introduction

Representation of the interior of the body has become more precise with the development of imaging techniques for biomedical exploration and diagnosis [1]. There are ultrasound scans, tomography, X-rays and magnetic resonance; all of them provide detailed images of the inside of our bodies. Obstetric ultrasounds are perhaps the images that have had the greatest social impact in the reproductive field [2,3], making it possible to identify the sex of the fetus and various risks during pregnancy. Today, these images continue to have great symbolic power for future mothers.

The technological precision of ultrasounds, according to Imaz [4], reinforces the concept of the fetus as an individual by connecting the power of the internal image of the body of the mother with/to the visual recognition of a differentiated dependent individuality—the fetus. This stands in contrast to the growing uncertainty women have about what happens inside their body during pregnancy and breastfeeding in terms of processes of internal contamination through the accumulation and transmission of environmental contaminants in foods, and their effects on the health of the fetus [5]. A wide variety of environmental chemicals is present in foods, from toxic metals to persistent organic pollutants (POPs). In particular, persistent toxic substances (PTSs), that include toxic metals and POPs, are chemical agents used in agricultural and industrial production that accumulate in the body at low doses over time, mostly through the consumption of food products containing animal fats [5]. Despite their lipophilic and bioaccumulative capacity in the body, PTSs are characterized by being perceived as a silent risk due to the short-term invisibility of the exposure and its consequences for health.

Toxic chemical exposure during pregnancy and the breastfeeding period is relevant as a health risk for women, fetus and babies in the present and future [6]. Scientifically, the risks to the fetus when pregnant women are exposed to certain toxic substances and PTSs are known. For example, with alcohol consumption, there is the risk of fetal alcohol syndrome [7]; with tobacco, fetal growth is restricted [8] and the endocrine, respiratory, cardiovascular and neurological systems are altered [9]; and lastly, with certain medications, such as thalidomide in the 1960s and more recently, valproic acid, genetic malformations occur [10].

However, although the risks of internal PTS contamination are not well-known among the general population, scientific findings are beginning to be widely disseminated, and pregnant and breastfeeding women are beginning to view PTSs as a potential risk for the development of the fetus and future health of the baby [11,12]. For example, in the area of food and diet, a large part of the dioxins that mothers ingest is through the consumption of dairy products, fish and meat, which can affect the health of the fetus through intrauterine exposure [13]. Various scientific studies focused on the exposure to PTSs have revealed their effects on the endocrine system [14,15] and neurotoxicity in pregnant women, babies and children during the growth stage [16]. Other effects of toxic metals on the reproductive system are also well known [17]. In order to prevent the harm of toxic environmental chemicals for human reproduction, the International Federation of Gynecology and Obstetrics published recommendations for health professionals [18].

In this article we analyze how pregnant and breastfeeding women represent the inside of their bodies and the way they think that the chemical compounds present in food are eliminated and accumulated, and how they are then transmitted to the fetus. In order to do this, we examine the different social perceptions of risk regarding the circulation of chemical compounds inside the body, and we use the qualitative research technique of body mapping, combining figures of the body with commentary, as well as food diaries and narratives from in-depth interviews.

Medical anthropology uses the qualitative technique of body mapping as a participatory method to help understand how the body and the effects of biomedical intervention on it are perceived. Considered a less intrusive instrument than a structured interview, it has been used with women from countries characterized by medical pluralism as a method to explore various ways of interpreting the body and biomedical interventions related to health.

The body acquires a central place in the representation of scientific ideas. Science and technology have often used images and figures to explain these complex ideas and communicate them to the public [19,20]. In this sense, we ask what representations are elaborated by pregnant and breastfeeding women to explain complex ideas, such as the perception of the risk of PTSs.

The use of this technique, at the individual or group level, implies that the researcher recognizes the existence of verbal difficulties in designating anatomical and other specific aspects of biomedical knowledge related to corporality. Therefore, its purpose is to incorporate figure to clarify ambiguities in the process of identification, attribution and anatomical classification. For example, in Zimbabwe [21] this technique was used to find out about local beliefs regarding conception and contraception in order to recognize differences between the explanatory models of women and health agents. However, the purpose was not to recognize their “ignorance” or to find out if what they knew was “correct,” nor what they said they knew, but instead, to explore the diversity of common perceptions and knowledge through incorporating this technique in group discussions. For Cornwall [22], the technique involved building bridges between local and biomedical knowledge and taking into account that the interaction of explanatory models involved recognizing different ways of knowing, conditioned by access to knowledge and determined by power relations.

One of the greatest risks of this technique is over-interpretation, as pointed out by Zaman et al. [23]. A figure can evoke an important element of the inner body that the informant would not have thought about unless the researcher had asked about it. In addition, it is the researcher who ultimately interprets the figure. Therefore, in our study, this potential bias has been compensated for by triangulating various techniques (interviews, focus groups, food diaries).

There are other applications of body mapping in the field of reproductive health, such as fetal growth or observing diet from the prenatal phase to the end of pregnancy [22], and sexual and reproductive health education. On the one hand, a recent study systematizing and revising the technique of body mapping as part of a participatory method applied to interdisciplinary research, treatment and education, observed its potential to produce and disseminate knowledge in educational, clinical, research and policy spheres in the field of health and disease [24]. The fact that participants have to draw body images, color, paint, decorate and share them involves a creative, reflective and at the same time, therapeutic process. On the other hand, the use of the body-mapping technique was applied to sexual education of young women to promote sexual subjectivity and wellbeing through multi-sensorial orientation [25]. This orientation enhanced the sensory perception of young women to construct body maps.

## 2. Material and Methods

We take into account data from the body-mapping technique applied to the study of pregnant and breastfeeding women’s figures of their body and the comments that accompany them on the mechanisms of transmission, accumulation and elimination of chemical compounds present in food. This technique was applied in an individual, free and autonomous way—through the making of food diaries—to better explore explanatory models for scientific and biomedical issues that are less known and not central to the doctor–patient relationship, which is the case of the risks of PTSs in food consumption.

This technique, complementary to other techniques used in the project, was not used as part of a participatory method, but as an instrument to explore the understanding these women had of the mechanisms involved in this internal pollution, which had previously been discussed in comprehensive interviews. In no case was this technique used to assess the level of scientific knowledge of the women, but rather to explore what they knew and how they thought about the mechanisms of internal pollution.

Once the in-depth interview was over, each woman participating in the study received a food diary to fill in, collected at their home after a few days. None of the women interacted with the other informants or with the researchers during the process of making the figures and explaining them. In this way, they avoided comparing what they knew with what others knew on this subject. This was a delicate issue that could generate uncertainty and even feelings of helplessness.

In one of the sections included on the fifth day of the diary, the women were asked to draw “the way that your body eliminates and accumulates ‘chemical compounds’ present in foods and how you can transmit these compounds to the baby”; following this, they were asked to explain the figure in writing. In this way, the data obtained from the figures were complemented with information obtained from their written responses, providing contextual information and written narratives.

Of the total of 111 women interviewed who participated in the interdisciplinary research, only 71 returned completed food diaries (Table 1, 40 pregnant and 31 breastfeeding women). All of them signed the informed consent, a mandatory requirement after the approval of the corresponding ethics committees.

There were 41 women, 21 who were pregnant and 20 who were breastfeeding, that completed the section in which they were asked to draw the way their own body transmitted, accumulated and eliminated the chemical compounds present in foods and the mode of transmission of these compounds to the baby. Of the 41 women, 23 completed the figure and the explanation (6 pregnant and 17 breastfeeding) and 16 (13 pregnant and 3 breastfeeding) did not complete the figure but did complete the explanation. Finally, two pregnant women completed a figure but without any explanation. In Table 2 below, the women’s occupations are also included, as this variable is significant for the analysis of the figures.

The act of figure (or not) with explanations on how these chemical compounds are eliminated, accumulated and transmitted involved a cognitive effort on the part of the women to answer the question and reflect on the knowledge they had about the effects of these compounds inside their bodies. They did not always draw and explain how the three processes occurred. The women who did not complete the figures explained that they were not good at figure.

Of the 23 women who drew and explained their figures in writing, 6 were pregnant and 17 were breastfeeding. With the exception of two women, one breastfeeding and one pregnant, who were unemployed at the time of the interview, all the others were working at the time the research was carried out in occupations corresponding to their level of education. As most of the women who drew a body were breastfeeding, it is striking to observe that most of their figures represent pregnant women, as we will see below. Breastfeeding was hardly represented in the figures, nor was the risk of transmission of chemical compounds through breast milk. Where breastfeeding was represented, they mostly drew the food consumed by the mother, as the study only included women breastfeeding during the first 6 months, before their babies were likely to be eating any solid foods. Among the 16 women who did not make figures but did write explanations, 13 were pregnant and 3 were breastfeeding.

In order to analyze the meanings from their figures and narratives and to understand the experiences of these women, we identified themes, patterns and topics to create codes and categories following the strategies of grounded theory [26,27]. The information was exhaustively systematized with ATLAS-TI -qualitative analysis software.

## 3. Results and Discussion

### 3.1. Body Mapping of Internal Pollution

The majority of the women who completed the section of the food diary where they were asked to make a figure and reflect on it worked in the health, education, services and industrial sectors. Accustomed to transmitting information to patients, students and customers, it is not surprising that they were the ones to make the most effort to complete figures and reflect on the internal pollution caused by chemical compounds.

Although the question focused on representing and explaining the three processes—elimination, accumulation and transmission (in that order)—the women most often made figures depicting the transmission of chemicals through the placenta and the umbilical cord. After that, and in order of importance, the figures most frequently depicted the elimination and transmission of chemical compounds in the same design. These were followed by figures that represented the circulatory dynamics of the three processes, and then, figures depicting accumulation and elimination together. There were very few women who drew representations of elimination or accumulation independently, in contrast to what we found in the case of transmission. Figures showing both accumulation and transmission were also not frequent. Regarding elimination, the women’s comments revealed certain difficulties in determining exactly what was eliminated and what could not be eliminated, and the elimination of toxins was confused with that of PTSs. Finally, regarding accumulation, we identified difficulties in knowing which organs and tissues these chemical substances were accumulated in. In addition, the women said in the interviews that they did not know how much time it took for these compounds to trigger harmful effects on health.

The article will take into account this order of representation and reflection. We begin first with how the body is drawn. This is followed by transmission and the centrality of the placenta and the umbilical cord; elimination and transmission; elimination, accumulation and transmission as a complex process; and finally, accumulation and elimination.

### 3.2. Representation of the Body: Separate Organs and Fluids in Movement

Most of the figures in the food diaries are of a transparent body showing the organs (stomach, lungs, liver, kidneys, intestines), and primarily, the digestive system. A previous study had already shown that food occupied a fundamental place in perceptions of the risk of contamination by synthetic chemical substances [28,29,30].

Often, the women drew a set of separate organs and parts, although sometimes linked by arrows, but without showing an integrated system. On the one hand, this kind of representation alludes to the mechanistic body model in which the most important element is physiology and anatomy, in other words, the form of the parts and the movement between them [31]. The mechanistic body model was flat, and the emphasis was placed on the particularity of the body machinery, its parts and mechanisms. On the other hand, although bio-medicine has developed a more complex and comprehensive knowledge about the exterior and interior of the human body, most people still do not have a good understanding of the physiological processes that organize various bodily functions, nor do they know how to represent the organs very well or place them inside the body.

Although this lack of knowledge about the inside of the human body (its organs and functions) affects the lack of anatomical precision in the figures, this does not imply that they combine different corporal models typical of popular knowledge [31]. In short, in this study, the women have an anatomical view of separate organs in the body or integrated into a system prefigured by a transparent body. The figures they draw include flat and neutral bodies, without reference to the female sex; bodies of mothers, sometimes with bulging bellies with a fetus inside, and breasts that allude to breastfeeding; bodily orifices with arrows indicating where chemical compounds enter and are expelled from the body; and maternal figures with emotional expressions (smiles) and social expressions (hair and clothing). Therefore, their figures depict organs separated from the body, autonomous bodies and bodies representing happy subjects.

In the case of this study, these bodies’ subjects are pregnant and breastfeeding women in relation to the fetus and baby. In an anthropological study on interpretations of the pregnant body in Basque society and the social representations of motherhood carried out by Imaz [4], three different “metaphors of the maternal body” were analyzed. The common denominator is that the pregnant body is defined in terms of a “body-for-another”: the fetus. According to this author, the differences between these metaphors are marked by a first reading of the body as a symbiosis between the pregnant woman and the fetus, a second reading in which the maternal body is parasitized or invaded by the fetus, and a third reading represented by the image of fetus as individual, in which the pregnant woman contains another dependent body and therefore, is represented as a split body [32]. These models overlap and combine in the reproductive imaginary. As we will see throughout this article, the combination of these metaphors is present in the figures of the women.

The figures contain many arrows and flows to represent the movement of chemical compounds as fluids and substances that enter and exit through the orifices of the body. The circulation of these substances usually converges in the digestive system. The arrows indicate the dynamism of transmission and the relationships between the exterior and interior of the body. Among the more graphic representations, there are figures that show bodies as a set of data; standing out among these are a pie chart, schemas, block diagrams and individual organs and organs connected by arrows. These graphic representations are usually used to show the three mechanisms of internal pollution. A body model more focused on the movement of fluids, which represented chemical circulation in a way similar to blood or placental circulation, contrasted with another more static and flat model represented by organs inside a body due to basic anatomical knowledge and images from X-rays.

Chemical compounds are difficult to represent graphically because in general there is a lack of knowledge about what they are and what the mechanisms of internal pollution are as well. They are usually represented in the figures with the name “chemical compounds” and an arrow that indicates the way they enter the body. To a lesser extent, they are identified with danger signs, such as skulls and traffic signs, and even with industrial baked goods and “contaminated” fruit.

### 3.3. The Transmission of Chemical Compounds: The Placenta and the Umbilical Cord

Women usually first represent the transmission of chemical compounds as a direct action from the mother to the fetus/baby. They think that what they eat could cause problems in growth and brain development. The placenta, the blood and the umbilical cord are the main transmission channels for chemical compounds, although we will see that not all women use the same body metaphors, nor do they all coincide in the way they explain maternal–fetal connections. Some of them are clear that the circulation of chemical compounds occurs through the umbilical cord; however, they may have doubts when reflecting on placental circulation. For example, one pregnant woman says:

“The chemical compounds we eat, I think they’re transmitted to the baby through the umbilical cord, like everything the mother eats and drinks. It’s possible that they’re also transmitted through the placenta, (but) I’m not clear about that” (Pregnant, university education, tourism sector).

This narrative contains two different metaphors, which are also related [4]. The metaphor of the “fused body”, common among pregnant women, is represented by the umbilical cord as a nexus symbolizing both the physical bond and the emotional dependence between both bodies—first united at a physical and vital level, and then connected after childbirth on an emotional level. Secondly, there is the metaphor of the “split body”, influenced by medical technology through the spread of sonograms and studies of the placenta; this has differentiated the pregnant woman and fetus into two distinct individuals who share the same body but with different needs and functions, and who are dependent on each other at the same time. Therefore, the relationship between the mother and the fetus becomes a potentially complex and risky relationship throughout the reproductive process. Hence in recent years, feminist research focused on the placenta is taking a different approach to examining the physical link and the symbolism of the pregnant woman–fetus relationship [32,33,34,35].

Only a few of the women think that these chemicals might not be transmitted to the fetus, with the placenta acting as a barrier: “I understand that the body is wise, and if it’s bad for the baby, it’s not transmitted” (Pregnant, economics degree, works as an executive secretary). However, none of the women have doubts about the consumption of alcohol, a substance they consider very dangerous for the development of the fetus and because of its effects on mental health. Medical advice on the health risks to the fetus when the mother consumes alcohol during pregnancy is very present in the women’s narratives. In the pregnancy monitoring protocol in Catalonia [36], the Department of Health discourages the consumption of alcohol during this period due to the risks to fetal development.

In the women’s figures, the fetus is less often drawn inside the body than separate from it. When they do draw the fetus inside the mother, the figure of the fetus is much more elaborate and occupies a central place. This centrality of the fetus is related to a recent technification in the process of the medicalization of pregnancy and progress that has been made in biochemical studies of the placenta, as mentioned above.

In general, the placenta is seen as a permeable membrane that actively places the mother and fetus in relationship through the circulatory system connected by the umbilical cord. In relation to placental circulation, one pregnant woman thinks that:

“In the case of pregnant women, the placenta is fundamental; it does the functions of all the organs of the future baby. Important: the blood of the fetus and the mother’s blood do not mix because the membrane that separates them lets the oxygen and nutrients pass, but not the rest. Along with the nutrients there are also all the additional substances (preservatives, dyes, metals...)” (Pregnant, university education, secondary school teacher).

**Figure 1:**
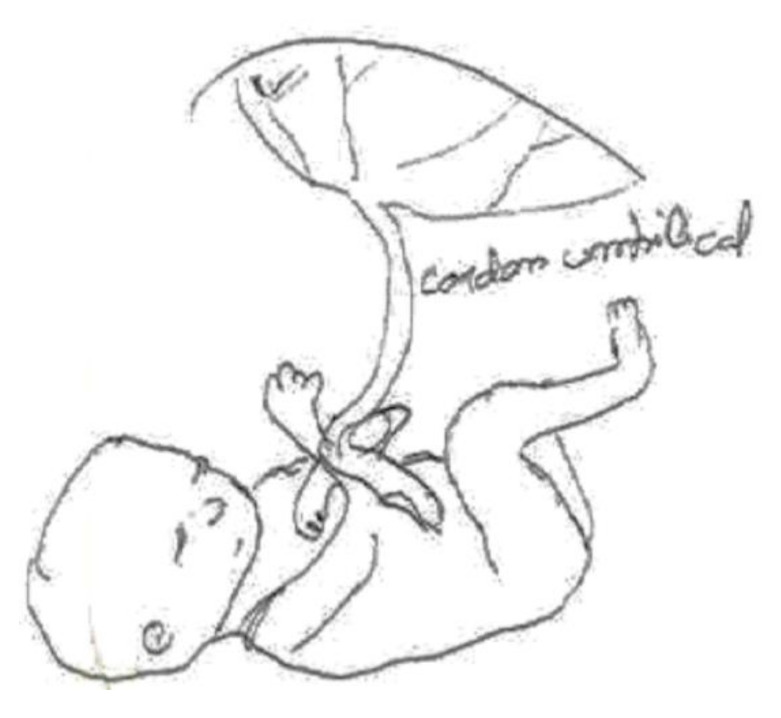
Breastfeeding, secondary education, employed in retail.

In the following Figure 1, we can see the connection between the fetus and the placenta through the umbilical cord. The pregnant woman’s body is absent. It emphasizes the function of the umbilical cord as the nexus connecting the placenta with the body of the mother.

Although the informant was breastfeeding, she preferred to draw transmission through the umbilical cord during pregnancy; she says at the bottom of the same figure: “Chemical compounds are transmitted to the baby through the umbilical cord.”

**Figure 2:**
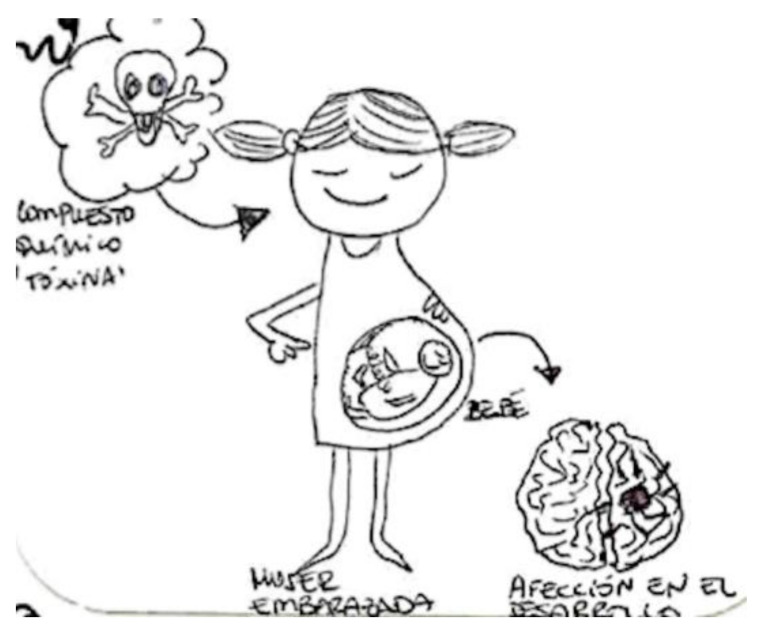
Breastfeeding, university education, surveyor.

In the next Figure 2, another breastfeeding woman preferred to draw the transmission of chemicals in pregnant women. This woman draws a smiling pregnant woman with a fetus inside and points out the danger, represented by a skull, with a phrase that says, “toxic chemical compound,” and with a design located in the lower part of the figure referring to the risk of disease in the development of the brain of the fetus.

Despite demonstrating in the figure that “chemical compounds reach the baby through the placenta after the mother consumes them,” in a previous interview, she had said the opposite—that the placenta acted as a filter and that almost nothing reached the baby. These ambiguities are found in other narratives in the study.

**Figure 3:**
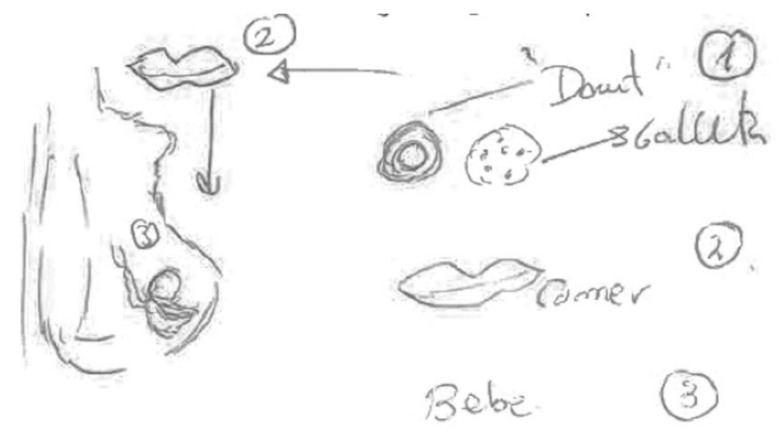
Pregnant, university education, employed in social services.

In the following Figure 3, completed by a pregnant woman, and which also shows chemicals being transmitted through the umbilical cord, the mechanism of transmission and the role of food in the process of contamination are highlighted. For this reason, she prefers to draw and point out the foods that most affect the health of the fetus and to highlight the mechanisms of transmission from the mother’s mouth, through the ingestion of food, through the body, and then to the fetus through the umbilical cord.

Food occupies a predominant place in internal pollution. In this reflection, a pregnant woman says that:

“The chemical compounds present in food are those such as mercury (usually found in fish such as tuna), pesticides (can remain in fruit and vegetables) and hormones (some animals are injected with hormones to accelerate their growth), etc [...] Many of the chemical compounds present in food can cross the placenta of pregnant women and reach the developing fetus, which could influence fetal development” (Pregnant, university education, town councilor).

Thus, eating is considered the main way that most chemicals enter the body, but in some cases, the women also include cosmetics and personal care products as dangerous substances that enter the body through the skin.

### 3.4. Elimination and Transmission: Some Substances Are Excreted and Others Are Transmitted

Transmission is also combined with the elimination of chemicals. According to the women in the study, elimination consists of the excretion of toxic substances from the body through the excretory organs such as the kidneys, the lungs, sweat glands, the liver and the intestines. Body orifices are the focus in the narratives regarding elimination. The risk comes from the substances that are not eliminated and remain in the mother’s body, thus potentially affecting the fetus. The orifices are therefore the entry and exit points that are most vulnerable to pollution [37].

**Figure 4:**
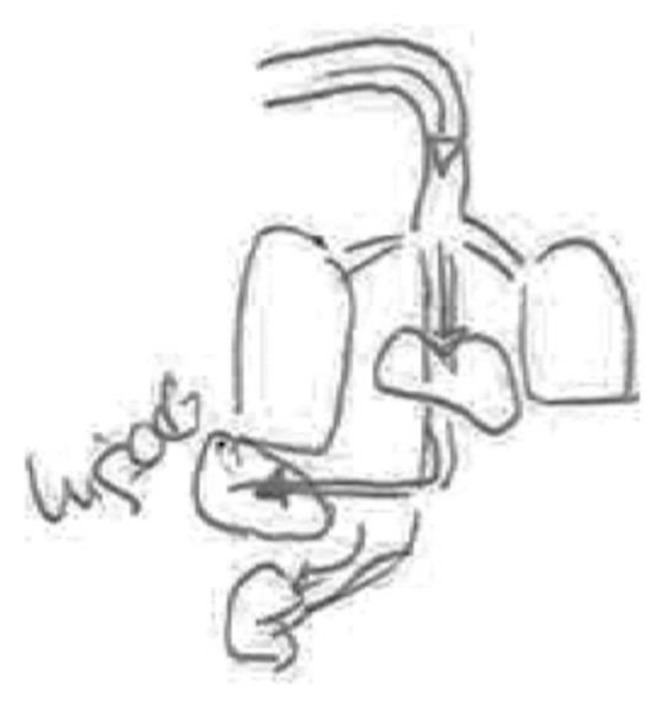
Pregnant, university education, nurse.

In the following Figure 4, a pregnant woman draws the organs without the body that contains them. The figure highlights the lungs, liver, stomach and kidneys. For her, the liver is the organ that metabolizes the chemicals, while the kidneys are the organs that eliminate them in part, with the remnants being transmitted to the fetus through the placenta.

Although the elimination of chemical substances is considered a part of the excretory processes of defecating, urinating and sweating, the women think that there is also a proactive way to eliminate these substances through certain practices to detoxify the body, such as eating organic or other specific foods [30], and through activities such as sports, hydration or massages. Some women confuse the elimination of chemical compounds with the elimination of toxins and attribute these practices to different processes. Thus, they believe that PTSs can also be eliminated through urine, sweat and feces, ideas that are partially supported by scientific knowledge. For example, some variety of PTSs are excreted by feces [38]. On a scientific level, it has been shown that the risk of PTSs can only be eliminated through reducing environmental exposure to them, requiring the implementation of specific administrative, policy and educational measures. Diet is considered the main source of internal contamination of PTSs [39,40]. Thus, actions must be maximized to regulate, control and prohibit exposure to certain PTSs during the reproductive cycle, applying the precautionary principle and carrying out preventive measures that stress greater control over these compounds in what we breathe, drink, and touch [41,42].

Some women believe that the ability to expel harmful substances from the digestive system is different in the mother and child. In the following narrative, a pregnant woman says that:

“I’m not an expert on the subject, but I think I read that through digestion adults can expel many harmful substances that children cannot, so it’s more important for children to eat food with the least amount of chemicals. In terms of how the baby is affected, as he or she eats and is fed by what the mother consumes, so it’s clear” (Pregnant, higher education, professor of Spanish literature).

**Figure 5:**
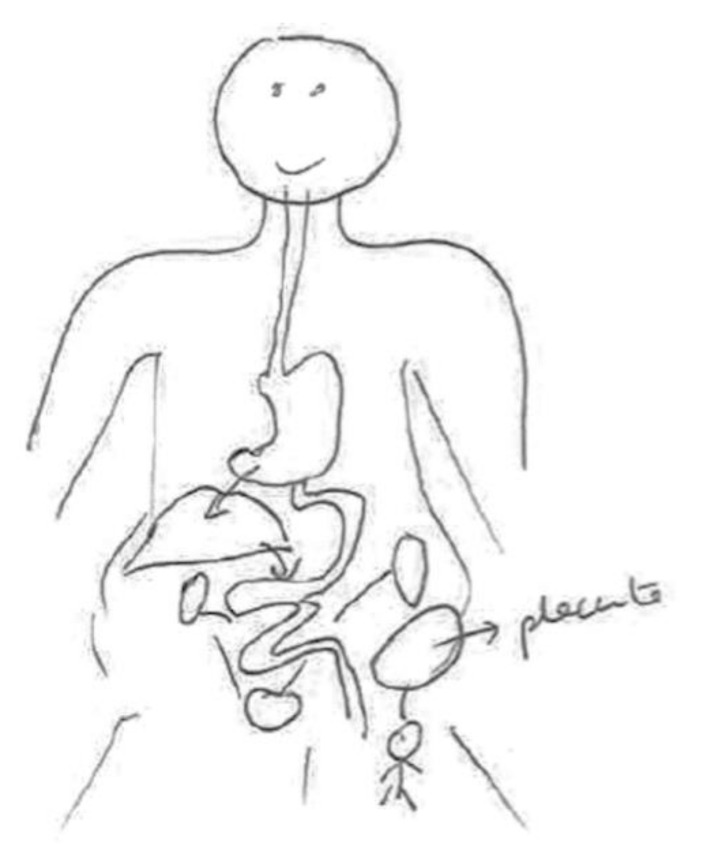
Pregnant, university education, pediatrician.

In the following Figure 5, the stomach, kidneys, liver, intestines and the placenta are shown. While filtration is thought to take place in the liver and kidneys, elimination takes place through the feces and urine. Some of the chemical substances can be transmitted from the blood to the placenta and thereby reach the fetus. Although this informant is a pediatrician, she is not sure if these substances are accumulated and does not know the health effects they might have. The only organ labeled in the figure is the placenta.

**Figure 6:**
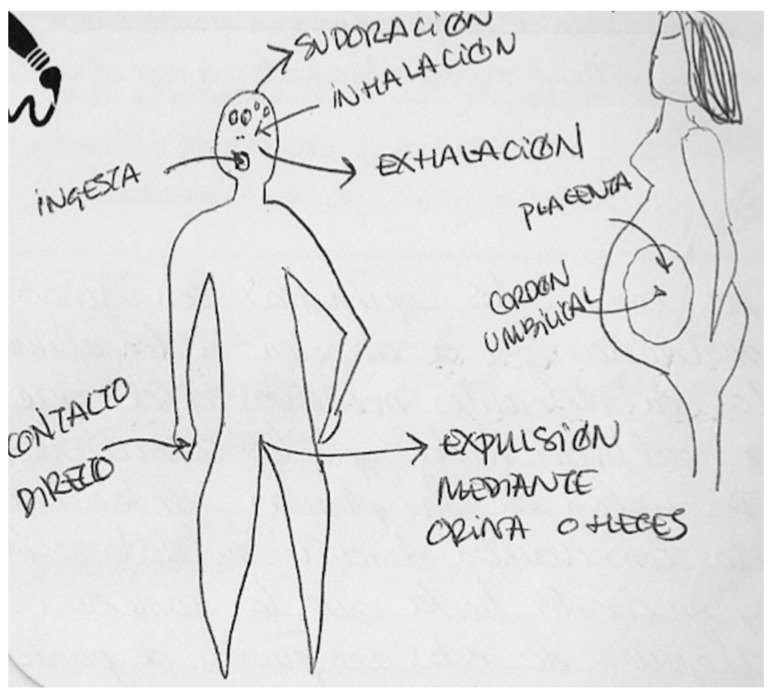
Pregnant, university education, architect.

In the next Figure 6, the informant draws two bodies, showing elimination on the left and transmission on the right. The first body shows the mechanisms prior to excretion, such as ingestion, inhalation and contact through the skin, and the last stage of secretion, such as sweating, exhalation, and expulsion through urine and feces. The second body shows the profile of a pregnant woman without the fetus, but with reference to the placenta and the umbilical cord. Both processes, elimination and transmission, are indicated by two arrows, one input and one output. When comparing this figure with the information previously provided in the interview, we see that this woman does not know if the chemical compounds are accumulated. In addition, while she was sure that transmission does occur during pregnancy, she claims that it does not happen during breastfeeding.

**Figure 7:**
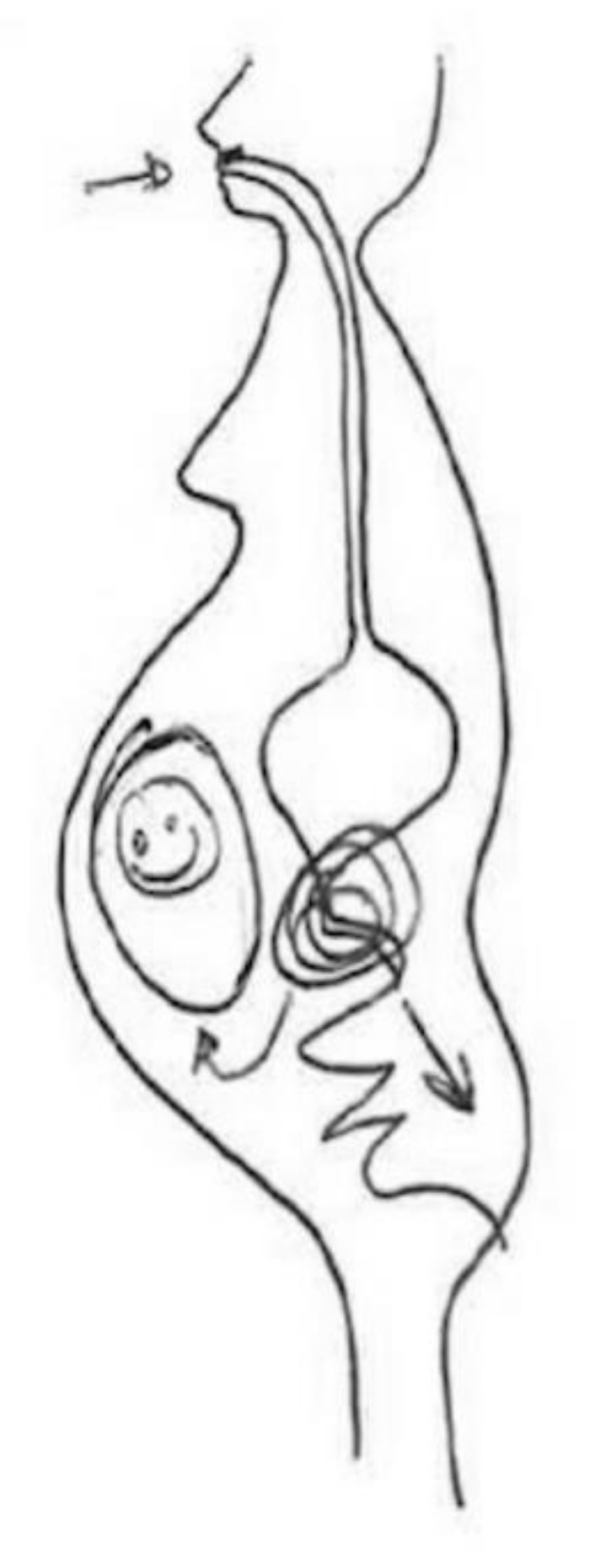
Pregnant, secondary education, employed in marketing sector.

These representations based on flows, indicating the entry and exit of chemical compounds through body orifices, are also found in the following Figure 7, but integrated in a single figure of the body:

The previous Figure 7 shows how the food we eat is absorbed by the digestive system. There are substances that pass through the intestines and excreted in the form of feces, and other substances that affect the fetus through the placenta. The idea of a body that knows how to eliminate what does not suit it through the digestive system seems clear, both in this figure and in the previous cases. However, the explanation of the figure refers to the circulation of substances that are also not suitable, but that remain in the body as chemical compounds.

### 3.5. Elimination, Accumulation and Transmission: Representation of a Complex Process

The representation of the three mechanisms of internal pollution are also usually prioritized in the figures of pregnant and breastfeeding women. In these figures the women represent and explain how these chemicals circulate inside the body. We see that their perspective is based on a model that envisions a circulatory movement of chemical compounds as if they were fluid.

**Figure 8:**
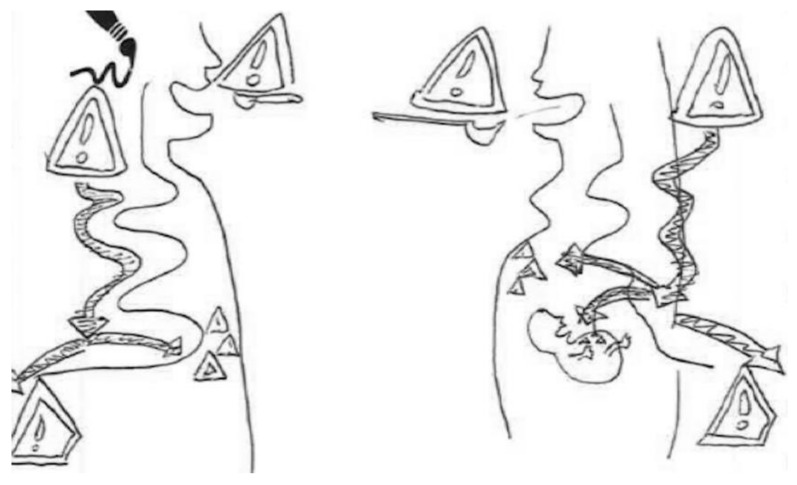
Breastfeeding, higher education, social worker.

In the following Figure 8 by a breastfeeding woman, two bodies are represented: one, in which we cannot say if it belongs to a man or a woman, and the other that shows a pregnant woman. Both use schematic elements to show the links between accumulation, elimination and transmission. In the upper part of the figure, there are two “traffic signs” indicating danger next to the mouth to indicate the risk of chemical compounds ingested in food. In the body on the left side, the circuit followed by the chemical substances is from the mouth to the rectum, passing through what is supposed to be the digestive system. In addition, we see that some substances accumulate dangerously in the stomach, and the others are eliminated. This is a good example in which different arrows indicate the movement of fluids and compounds in the body. In the second body on the right, the circuit is the same, except that some of these chemicals can be seen to be transmitted to the fetus. Based on the data extracted from the interview, this woman believes that the accumulation of these substances occurs when large quantities are ingested, as she considers the body is unable to eliminate all of them. For her, the smaller size of the fetus indicates that it will receive fewer toxic substances. Finally, she believes that exercise is a way to help eliminate some of these toxins, an example of confusing toxic elements with toxins. In the narrative that accompanies the figure, the woman says the following:

“Chemical compounds enter the body through food or the air we breathe. The body expels as much as it can through the usual channels (feces, urine, sweat...). But there is always something that remains in the body, which can accumulate in pregnant women. These compounds also reach the baby since it’s fed with what the mother eats.”

**Figure 9:**
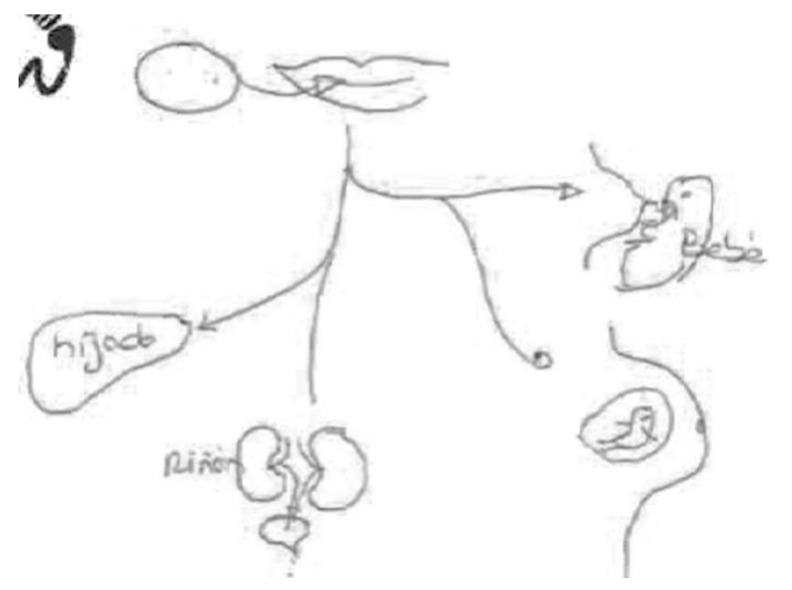
Breastfeeding, university education, doctor.

In the following Figure 9, a breastfeeding mother opts for a schematic representation of separate organs without placing them inside a body. There is a mouth, a liver, kidneys, a fetus and a baby sucking, all connected, again, by arrows. The arrows are supposed to link a bodily system of fluids—blood and milk—through which the chemicals circulate:

“The mother ingests the chemical compound, and these are eliminated by the liver or kidneys, or accumulated in fat, hair, etc. Chemical compounds travel through the blood of the breast. If she is pregnant they go directly from the mother’s blood to the baby, and in the case of a newborn, they go from the mother’s blood to her milk and to the baby who is nursing.”

**Figure 10:**
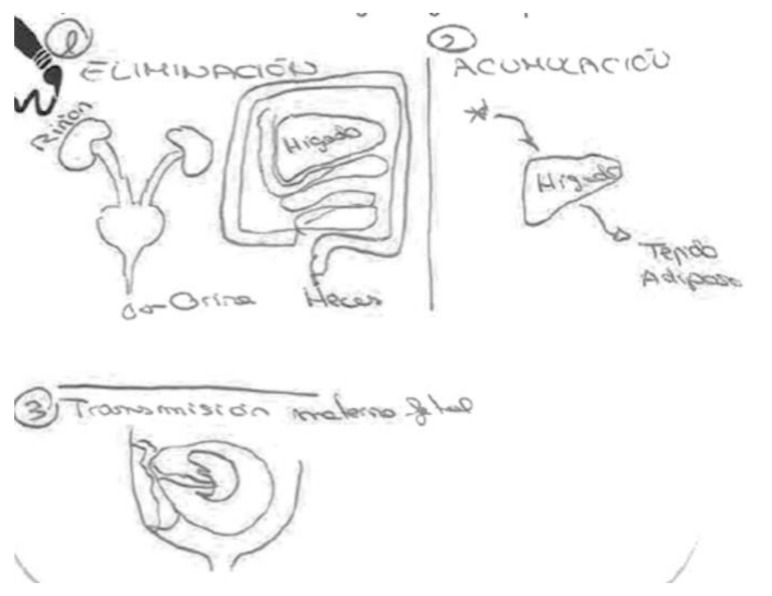
Breastfeeding, university education, nurse.

In another Figure 10, a breastfeeding mother also chooses to draw three separate processes. The organs are integrated into the renal, digestive, hepatic and reproductive systems. In addition, the organs are labeled the following: kidney, liver, adipose tissue, and fluids such as urine or feces. The fetus, the placenta and the umbilical cord are represented without being labeled.

The explanation with the figure is also schematic and presented in three stages:

“Elimination: 1. Mostly eliminated through urine. Some through feces. To a lesser degree, through breathing or sweating?

Accumulation: 2. Metabolized by liver and accumulated in adipose tissue.

Transmission: 3. Through the umbilical cord”

**Figure 11:**
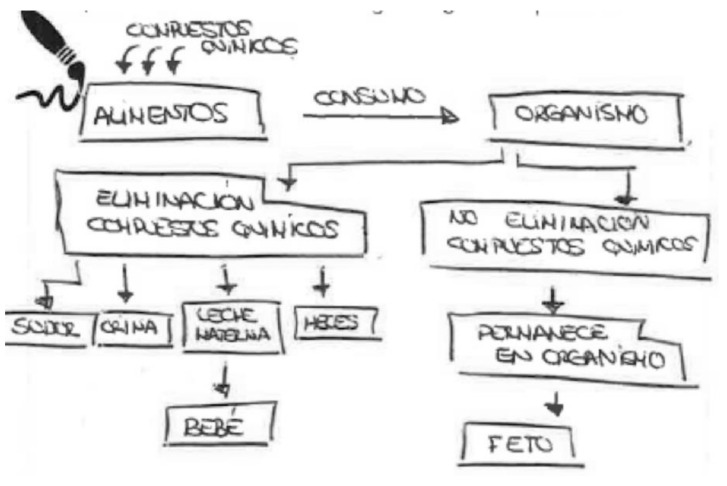
Pregnant, university education, nurse.

In the following Figure 11, a block diagram is chosen to explain the three mechanisms. The information is very similar to the previous one, although there is no body represented.

This schema is explained very well in the following description:

“Chemical compounds are added to food. We consume this food. Once consumed, we may or may not eliminate these chemical compounds. Among the elimination pathways, we have elimination through breast milk, which would pass to the baby through breastfeeding. The rest of the chemical substances not eliminated will pass to the fetus through placental circulation”.

**Figure 12:**
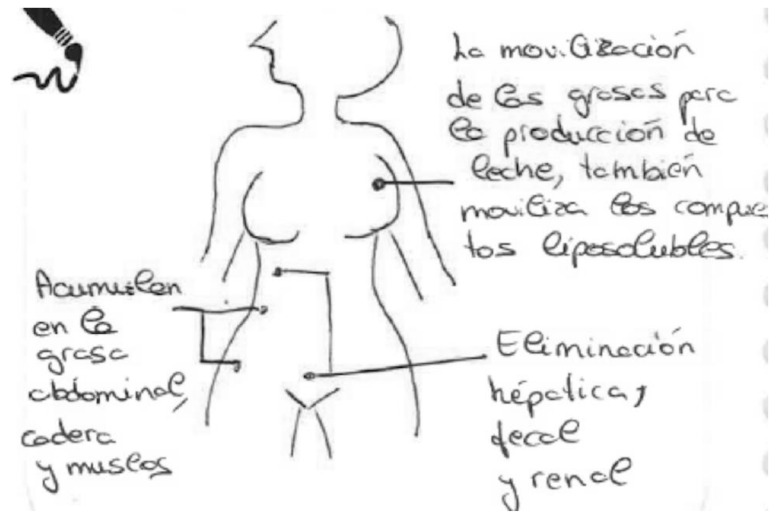
Breastfeeding, university education, secondary school teacher.

The next Figure 12, completed by a breastfeeding woman who worked in a medical school analyzing chemical compounds, shows the body of a woman without organs. The arrows indicate the three processes. The production of fats and breast milk plays a fundamental role in the accumulation process due to the fat-soluble nature of the chemical compounds. First in the figure it says that “the mobilization of fats for the production of milk also mobilizes fat-soluble compounds [...] (which) accumulate in abdominal fat, the hips and thighs [...] and (that) elimination is hepatic, fecal and renal.” In the explanation attached, she expands on this:

“Compounds enter through our diet. They’re processed in the intestines, liver; some of them will be eliminated in the feces. Others will be carried in the blood to the fatty tissue (being fat-soluble compounds, they will be stored). Others can pass to the renal pathway and be eliminated in the urine. When fat is used for milk production, they will be present in breast milk.”

**Figure 13:**
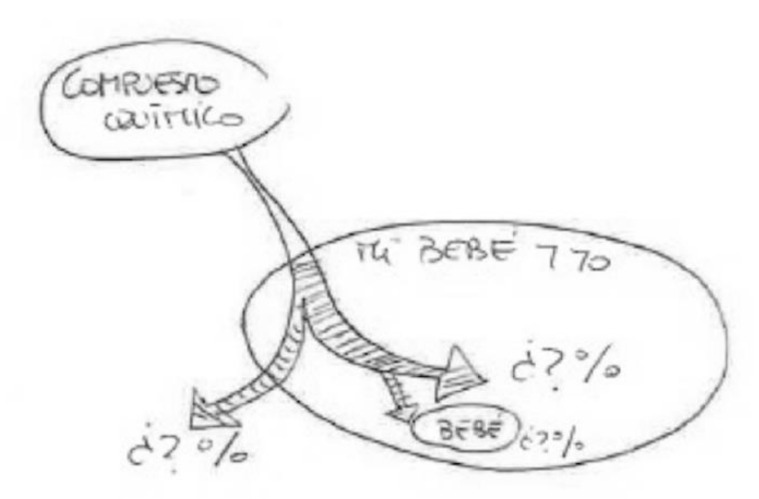
Breastfeeding, university education, veterinarian.

The women know little about the amount that accumulates in the body and how much of it could end up causing diseases such as cancer. Very few allude to the bio-cumulative nature of chemical compounds. In the following Figure 13, a conceptual map is used to explain the entire process of elimination, accumulation and transmission. Emphasis is placed on the lack of information about the percentages of chemicals entering, remaining and leaving the body:

“Of the chemical compounds that I ingest, I think that some of them—I don’t know what%—I eliminate, along with the rest of the “things” that my body doesn’t want. And the other% remains, and part of it passes to the baby. I think only a small% remains in my body.”

### 3.6. Accumulation and Elimination of Chemical Compounds

Ideas about accumulation are vague, not homogeneous and not very concrete. They are usually related to the risk of disease and, especially, the possibility of chemical compounds being carcinogenic. However, accumulation is more represented in relation to elimination than being explained separately.

The women think that processed foods contain more chemicals and tend to accumulate more often in the body. They know that some proportion of the chemicals accumulate, and others are eliminated, but the reason why and exactly where is not known, although women prefer to place them—as already mentioned—in the digestive system, the organs and also in fats. At the scientific level, it is known that PTSs accumulate mainly in adipose tissue [38,43].

**Figure 14:**
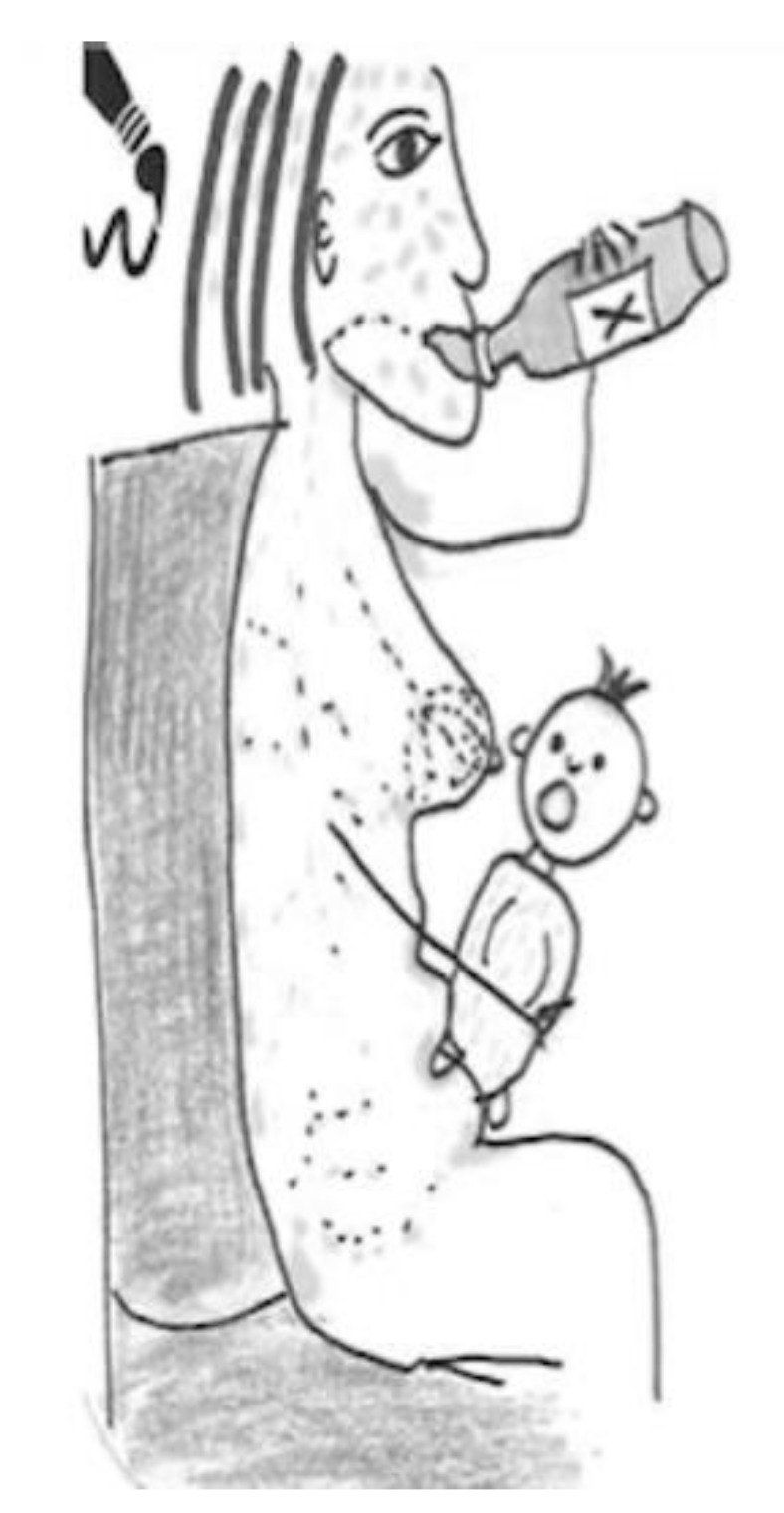
Breastfeeding, university education, primary school teacher.

In the following Figure 14, we see a pregnant woman and the process of eliminating chemical compounds and accumulation. In the narrative, it is pointed out that:

“Even though I know it’s not like that, I always think about what I drink and how it goes straight to the breasts, then it sticks to the walls of the body (the veins, intestines), and what doesn’t stick is eliminated in the feces or sweat. In proportion to the size of the baby, a lot more passes on to him or her, and it’s harder to eliminate.”

A new idea appears: chemical substances can stick to the walls of the veins, a process similar to the image many have of cholesterol.

**Figure 15:**
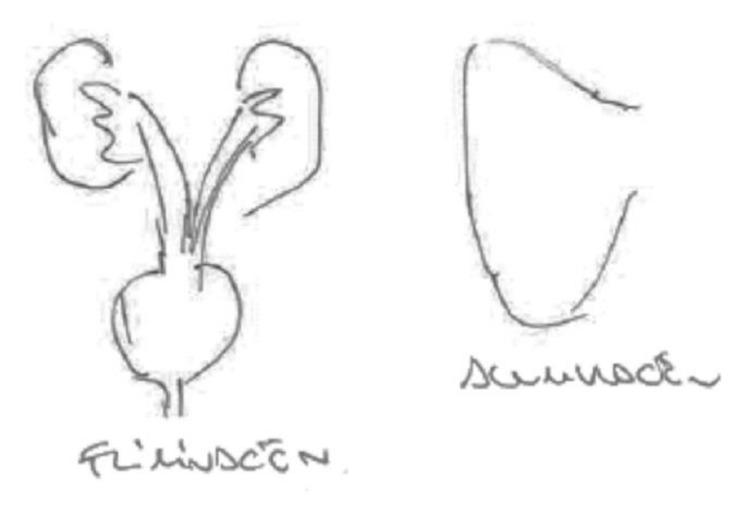
Breastfeeding, university education, doctor.

In the same way as we have seen in the other sections, accumulation can be represented in the organs separated from the body. In the following Figure 15, accumulation occurs in the liver and elimination through urine. In the interview, this participant says she assumes that the amount that is eliminated depends on the person, as well as on the amount that is accumulated, and it passes into the blood based on the susceptibility of each individual.

**Figure 16:**
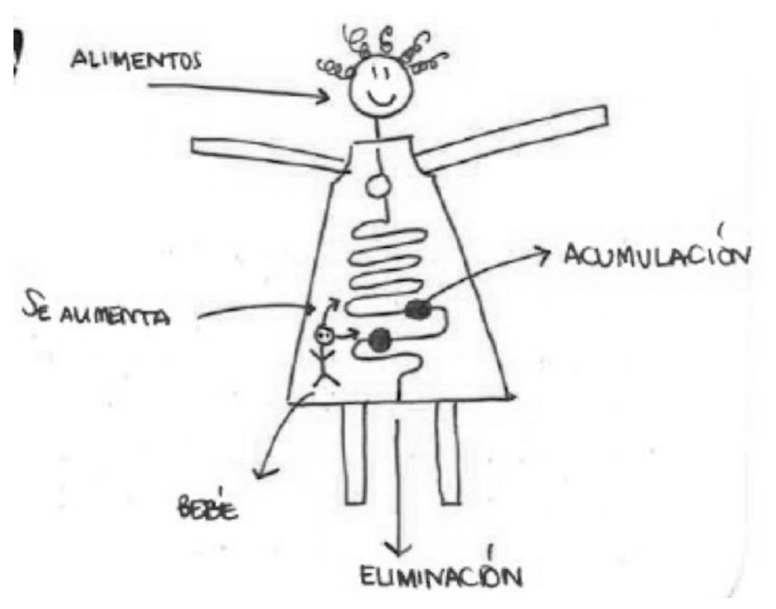
Breastfeeding, secondary education, sealing teacher.

The following Figure 16 is of a doll surrounded by arrows indicating the processes of accumulation and elimination in the intestines. The narrative emphasizes that “the food ingested by the mother is partly eliminated and partly accumulated. The baby is fed by accumulation (positive and negative) of these foods from the mother’s body.” There is a simple figure of a fetus with an arrow to indicate the act of eating and the process of accumulation.

## 4. Conclusions

Chemical compounds are difficult to represent. The women in our study imagined them as substances mixed in their blood and breast milk, or as fluids of an unknown nature circulating inside the body, with the ability to adhere to the walls of the veins and arteries and to be partially eliminated through feces and urine. We found that there are no significant differences regarding risk perception between pregnant and breastfeeding women and, for both, the period of greatest concern for exposure was during pregnancy. They do not know in what quantities chemical compounds become dangerous, nor exactly how or where they accumulate. What most of our informants do seem to understand is the ability of chemical compounds to be transmitted at the maternal–fetal level through the placenta, thereby affecting fetal development.

We found that there is no comprehensive representation of the inner body or a tendency to sexualize the external image of the body, although some participants did not forget to humanize their images with faces of smiling women. This positive appearance of the bodies makes us assume the representation of a subject happy to be pregnant, an image that is in contrast to the narratives of women concerned about the risks of internal pollution. Influenced by a mechanistic model of the body, they prefer to graphically objectify the inner body, as well as to separate it into parts and mechanisms when it comes to representing this type of pollution.

The lack of a coherent scientific communication, as well as social and personal experience about the symptoms related to this pollution, influence the difficulties that the women in our study have when linking this knowledge with their personal experience. Despite these difficulties, they recognize that exposure to synthetic chemical substances could have risks for the health of the fetus, and for that reason, they try to represent and present the knowledge they have, although they have doubts about it. The main concern of these women is the process of the transmission of chemical compounds. Their representations are focused on the fetus, the role of the placenta and the umbilical cord in the maternal–fetal relationship. Although some mothers have some doubts about the transmission of PTSs and the risks that this involves, most think that there is a process of connection and differentiation in the relationship between the mother and fetus. This scientific process, known as “placentation” [33] and the “placental economy” [32,34,35], places the biological contribution of the placenta at the center of the debate, and situates it as a relational organ through which functions and materials circulate between the mother and the fetus. The maternal–fetal exchange through the placenta becomes a central issue in immunological science [34], but also in regard to internal pollution from PTSs [44,45]. While the placenta protects the fetus from certain dangers, the mothers recognize that it cannot safeguard the fetus from all of them, and PTSs represent one of these dangers.

Bodily orifices are at the center of the participants’ narratives in regard to the process of elimination. They are the input and output channels that are most vulnerable to pollution [37]. For the women in this study, the chemical compounds that are eliminated have a liquid and solid texture. The confusion between toxins and PTSs means that fewer references are made to scientific explanations and measures to reduce or avoid exposure. In addition, many think that detoxifying their bodies with organic foods is a practice that will help to eliminate them.

Finally, the accumulation process is less represented and more difficult to concretize for the women in this study. Although there is scientific documentation on the effects of the bioaccumulation of PTSs [14,15], this knowledge is scattered and, therefore, the women have doubts about disease risk factors, such as amounts of toxicity, the place of accumulation and individual susceptibility.

A limitation of the present study is that although we did not target any specific group of women for participation, a large proportion of participants were women concerned about food risks and who had university educations. Thus, in the future, further research will be necessary to reflect on how we could achieve a more heterogenous sample and thereby generate a better understanding of risk perception among different groups of pregnant and breastfeeding women.

In short, the body mapping technique has allowed us to examine knowledge about internal pollution, a little-known process in society. Thanks to these pregnant and breastfeeding women, who made an effort to represent and reflect on these new risks, this study shows that scientists and obstetricians need to collaborate with women in order to better understand and publicize the risks of internal pollution. These results might help the development of public health campaigns as well as the adaptation of messages from health services to the pregnant and breastfeeding population, and, in this way, also improve the health of newborn children.

## Figures and Tables

**Table 1:** Socio-demographic characteristics of the women who completed food diaries.

	Pregnant Women	Breastfeeding Women
**Education level**		
Primary education	3	0
Secondary education	11	8
Higher education	26	23
**Age**		
20–29 years old	3	2
30–39 years old	33	23
Over 40	4	6
**Family structure**		
Extended family	0	1
Single parent family	1	0
Nuclear family	40	29
**Number of children**		
1 child	18	15
2 children	18	12
3 or more children	4	4

Source: By authors, 2016.

**Table 2:** Socio-demographic and socioeconomic characteristics of the women who completed the activity with or without figure.

	Pregnant Women	Breastfeeding Women
**Education level**		
Primary education	0	0
Secondary education	2	2
Higher education	19	18
**Age**		
20–29 years old	0	2
30–39 years old	21	16
Over 40	0	2
**Family structure**		
Extended family	0	0
Single parent family	0	0
Nuclear family	21	20
**Number of children**		
1 child	8	9
2 children	12	10
3 or more children	1	1
**Occupation**		
Educational sector: professors, teachers	4	5
Health care sector: nurses, doctors	4	4
Service sector: tourism, retail, hairdresser, communications, public administration, insurance	10	6
Industrial sector: architecture, engineering and interior design	1	3
Other sectors: veterinarian, lab technician	1	1
Unemployed	1	1

Source: By authors, 2016.
